# Health Services Utilization in China during the COVID-19 Pandemic: Results from a Large-Scale Online Survey

**DOI:** 10.3390/ijerph192315892

**Published:** 2022-11-29

**Authors:** Xia Wei, Haowen Yuan, Yan Sun, Jiawei Zhang, Qingbo Wang, Yaqun Fu, Quan Wang, Li Sun, Li Yang

**Affiliations:** 1 School of Public Health, Peking University, Beijing 100191, China; 2 Department of Health Services Research and Policy, London School of Hygiene & Tropical Medicine, London WC1H 9SH, UK; 3 Brown School, Washington University in St. Louis, St. Louis, MO 63130, USA

**Keywords:** health services, access, health seeking behavior, COVID-19, chronic disease

## Abstract

Timely access to essential health services is a concern as COVID-19 continues. This study aimed to investigate health services utilization during the first wave of the pandemic in China. A cross-sectional online survey was conducted using a self-administrated questionnaire in March 2020. Descriptive statistics and logistic regression were used for data analysis. A total of 4744 respondents were included, with 52.00% reporting affected services utilization. Clinical testing (68.14%) and drug purchase (49.61%) were the most affected types. Higher education level, being married, chronic disease, frequently visiting a provincial medical institution, spending more time on pandemic-related information, perception of high-risk of infection, perception of large health impact of the pandemic, and anxiety/depression were significant predictors for reporting affected services utilization. For the 431 chronic disease respondents, 62.18% reported interruption, especially for drug purchase (58.58%). Affected health services utilization was reported during the first wave of the pandemic in China, especially for those with higher education level, chronic diseases, and COVID-19 related concerns. Enhancing primary healthcare, use of telehealth, extended prescription, and public communication were countermeasures undertaken by China during the rapid rise period. As COVID-19 progresses, the changing disease characteristics, adapted health system, along with enhanced public awareness/knowledge should be considered for the evolution of health services utilization, and further investigation is needed.

## 1. Introduction

Coronavirus Disease 2019 (COVID-19) has led to substantial disease and economic burden worldwide. As a highly infectious disease, it may lead to further pneumonia and complications, such as acute respiratory distress syndrome, which is potentially life-threatening [1]. The emergence of the Omicron variant has induced rapid increase in the confirmed cases since the end of 2021 [2]. There have been approximately 620 million confirmed cases globally as of 14 October 2022 [3], among which approximately 0.25 million cases were reported in mainland China [4]. In response to the outbreak, a mix of measures, including non-pharmaceutical (hygiene regulations, social distancing, test-trace-isolation, lockdown) and pharmaceutical (vaccination and drug therapy) interventions, have been undertaken by many countries. As for China, it has entered the normalization period of COVID-19 prevention and control after the successful curb of the first wave of the outbreak in Hubei province by comprehensive measures [5,6].

As the COVID-19 pandemic continues to impact health systems globally, timely access to essential health services is still a concern [7,8,9]. The countermeasures to suppress the transmission of COVID-19 may make traditional forms of service delivery, such as screening, surveillance, prescription, and outpatient surgery, delayed or capacity-reduced, causing possible interruption to the routine care for other diseases [10]. Individuals with chronic diseases need to access health services on a regular basis, and they are also at higher risk of severe COVID-19-related illness and death [11,12,13]. The service deferrals may have a potential impact on their health in the long-run in case of complex complications.

A rapid survey by the World Health Organization (WHO) showed that the prevention and treatment services for non-communicable diseases were significantly impacted since the pandemic began [14]. Cancer screening [15,16], testing, medication, hospitalization for cardiovascular disease and diabetes [8,9,10,16], and mental health services [17,18] were found to be disrupted by the pandemic. Patient questionnaire survey or electronic medical records analysis were the predominant ways to capture these changes. The extent of the impact of the pandemic on health services utilization varied across studies, due to the heterogeneity in the type of health services, health facilities, and disease area. However, all of them highlighted that efforts were needed to guarantee timely access to essential health services during the pandemic.

In China, the number of patients with chronic disease has reached around 480 million [19], implying a need for massive routine health services. Significant decline in health services utilization was reported during February 2020 in mainland China, using routine health information system data [20] or healthcare transaction data [21]. Another two studies [7,22] also found a reduction in medical consultations among the general population or older adults with multimorbidity in Hong Kong, China, using data from telephone surveys or a medical record system. More evidence on the health services utilization during the pandemic is needed for the Chinese setting, especially from the patient perspective. This study aimed to investigate patterns of self-reported health services utilization and related factors during the first wave of the COVID-19 pandemic in China, to provide possible insights for ensuring essential health services utilization as the pandemic progresses.

## 2. Materials and Methods

### 2.1. Study Population

A cross-sectional survey was carried out from 1 March to 31 March 2020 to investigate the health services utilization pattern during the first wave of the COVID-19 pandemic in China (January to February 2020). Due to concerns regarding infection and movement restrictions, the investigation was conducted through an online survey platform. Chinese people aged 16 years or older who consented to participate were eligible for this study. Voluntary sampling was used to recruit participants, predominantly through social media (e.g., WeChat 7.0.10) in China.

Respondents first read an informed consent statement after clicking the survey link, which detailed the objectives of the study and that their participation was completely confidential and voluntary. Respondents need to answer a yes–no question to confirm their willingness to participate before proceeding to complete the online survey. Only anonymized data of the respondents were collected, thus not containing any personally identifiable or sensitive information. As an anonymous and voluntary online survey, this study was exempt from institutional review oversight.

### 2.2. Data Collection

A structured self-administrated questionnaire was developed. Sociodemographic variables, including gender, age, education level, marital status, employment status, and other information, along with health conditions and COVID-19 related concerns, were collected. Respondents also answered questions about the impact on their health services utilization and the type of affected health services during January to February 2020. Specially, respondents with chronic diseases (identified from the question ‘have you been diagnosed with chronic diseases?’) further answered questions about services utilization and expenses of outpatient and inpatient visits before and during the pandemic. The online survey took approximately 10–15 min to complete.

### 2.3. Statistical Analysis

Data analysis was performed using STATA version 17 (StataCorp LLC, College Station, TX, USA). To improve the quality of the online survey, we reviewed questionnaires and excluded those with arbitrary answers or missing responses. Baseline demographics were calculated using descriptive statistics. Means with standard deviations or medians with interquartile ranges were calculated for continuous variables, while frequency and percentage were calculated for categorical variables. Percentages indicating affected health services utilization were calculated based on an affirmative response to: ‘Has your health services utilization been affected during January to February 2020?’ divided by the overall respondents. Chi-square test was used to compare the difference in health services utilization for categorical variables, and *t*-test or Mann–Whitney tests for continuous variables. Binary logistic regression analysis was applied to predict the factors associated with reporting affected health services utilization including all variables. For respondents with chronic diseases, we further compared their health services utilization and related medical expenses before and during the pandemic using Wilcoxon signed-rank test [23]. The significance level was set at a = 0.05, and all tests were 2-tailed.

## 3. Results

### 3.1. Characteristics of the Study Population

Out of 5504 respondents, 4744 (86.19%) were involved in the final analysis. The survey was conducted nationwide. Respondents from Shanxi (N = 876, 18.47%), Hebei (N = 424, 8.94%), and Beijing (N = 401, 8.45%) ranked the top three, with less than five respondents from Xizang and Qinghai each. Demographic characteristics of the study population are summarized in Table 1. The mean age of the respondents was 31.88 ± 11.44 years. 2425 (51.12%) of the respondents were female, 2940 (61·97%) had college education, 3072 (64.76%) were currently employed, 2674 (56.37%) were married, and 2804 (59.11%) resided in the urban areas. Further, 76.85% of the respondents reported their annual household income was above 50,000 RMB in 2019.

A total of 3510 (74.00%) respondents reported annual household medical expenses higher than 1000 RMB in 2019, and 431(9.09%) respondents reported having at least one chronic disease. The frequently visited medical institution of the respondents included township health center (15.16%), community health center (16.51%), county-level medical institution (25.97%), municipal medical institution (27.61%), and provincial medical institution (14.46%).

For the COVID-19 related concerns, 3154 (66.48%) respondents spent more than one hour per-day browsing information related to the pandemic, 2027 (42.74%) respondents perceived high-risk of contracting COVID-19, and 2800 (59.02%) respondents believed that the pandemic had some/significant impact on their health. More importantly, 1991 (41.97%) reported occasional anxiety/depression due to the pandemic, while 442 (9.32%) reported frequent anxiety/depression symptoms.

### 3.2. Patterns of Health Services Utilization and Related Factors

**Figure 1:**
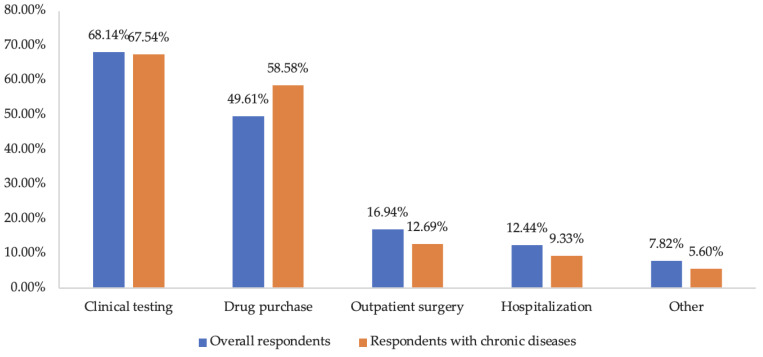
Types of health services affected during January to February 2020.

A total of 2467 (52.00%) respondents reported affected health services utilization. Clinical testing (68.14%) and drug purchase (49.61%) were the most affected types (Figure 1), which were mainly due to cold or fever (39.44%), oral diseases (14.66%), and digestive system diseases (4.84%). The reporting of affected health services utilization was significantly associated with higher education level, being married, residing in urban areas, higher annual household medical expenses in 2019, having chronic disease, spending more time on pandemic-related information, perception of higher risk of infection, perception of larger health impact of the pandemic, and having anxiety/depression during the pandemic in the univariate analysis (*p* < 0.05, Table 1).

After logistic regression analysis (Table 2), education level of middle high school (OR = 2.42; CI: 1.23, 4.73; *p* = 0.010); senior high school (OR = 2.17; CI: 1.11, 4.22; *p* = 0.023), undergraduate or higher (OR = 2.06; CI: 1.06, 4.02; *p* = 0.034) (vs. primary school or below); being married (vs. non-married, OR = 1.28; CI: 1.08, 1.53; *p* = 0.005); having chronic disease (vs. without, OR = 1.27; CI: 1.00, 1.62; *p* = 0.048); frequently visiting community health center (OR = 1.29; CI: 1.03, 1.62; *p* = 0.029) and provincial medical institution (OR = 1.34; CI: 1.03, 1.74; *p* = 0.029) (vs. township health center); spending less than one hour per-day browsing pandemic related information (OR = 1.33; CI: 1.07, 1.65; *p* = 0.011) and more than three hours (OR = 1.34; CI: 1.09, 1.66; *p* = 0.006) (vs. irregular time); perception of slightly high risk of infection (OR = 1.43; CI: 1.16, 1.76; *p* = 0.001), fairly high risk of infection (OR = 1.63; CI: 1.30, 2.05; *p* < 0.001), very high risk of infection (OR = 2.09; CI: 1.60, 2.73; *p* < 0.001) (vs. perception of very low risk of infection); perception of some health impact of the pandemic (OR = 3.14; CI: 2.72, 3.62; *p* < 0.001) and significant health impact (OR = 6.24; CI: 4.98, 7.82; *p* < 0.001) (vs. perception of nearly no health impact); reporting occasional anxiety/depression during the pandemic (OR = 1.39; CI: 1.21, 1.59; *p* < 0.001) and frequent anxiety/depression (OR = 1.80; CI: 1.38, 2.34; *p* < 0.001) (vs. no anxiety/depression) were significant predictors of reporting affected health services utilization.

### 3.3. Health Services Utilization among Respondents with Chronic Diseases

A total of 431 (9.09%) respondents reported having at least one chronic disease, with a mean age of 41.12 ± 13.65 years. Among these respondents, hypertension was the most common disease (268 respondents, 62.18%), followed by diabetes (77 respondents, 17.87%) and chronic renal disease (74 respondents, 17.17%). Moreover, 62.18% of the respondents with chronic diseases reported disruption in health services utilization. They also reported larger impact on drug purchase compared to the overall respondents (58.58% vs. 49.61%) (Figure 1).

**Figure 2:**
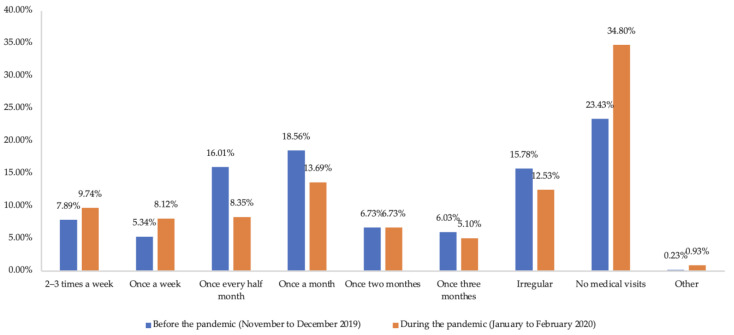
Frequency of outpatient visits in chronic diseases respondents before and during the COVID-19 pandemic.

For outpatient health services (Figure 2), there was a noticeable increase (11.37%) in chronic diseases respondents who did not receive any outpatient health services during the pandemic compared to before the pandemic (January to February 2020 vs. November to December 2019). Decline was also found in respondents who sought outpatient health services once every half month or less frequently during the pandemic. However, the proportion of respondents seeking outpatient health services once a week or more frequently went up a little bit during the pandemic. Moreover, statistically significant decreases were identified in all types of outpatient expenses, including total medical expenses, out-of-pocket medical expenses, drug expenses, and out-of-pocket drug expenses, when comparing January–February 2020 with November–December 2019 (Table 3). Further, 45 respondents (10.44%) received inpatient health services due to chronic diseases in 2019, but only 13 respondents (3.02%) during the first two months of the pandemic. Notably, 28 respondents reported early discharge as a result of the pandemic. Due to the limited number, we could not compare the inpatient expenses before and during the pandemic in respondents with chronic diseases.

**Figure 3:**
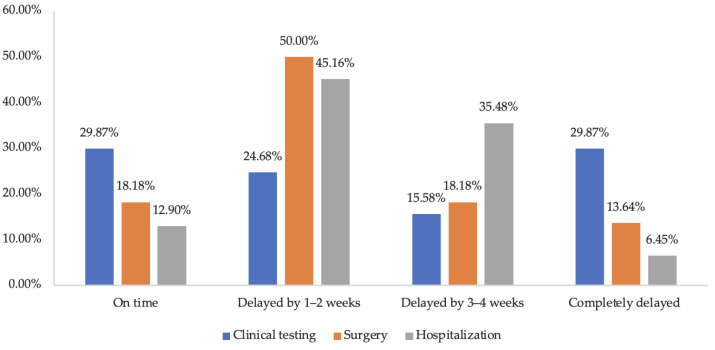
Health services appointment in chronic diseases respondents during the COVID-19 pandemic.

For appointed health services, 77, 44, and 33 of the 431 respondents with chronic diseases made appointments for clinical testing, surgery, and hospitalization before the pandemic, respectively. Among these health services (Figure 3), clinical testing had the highest rate of complete delay (29.87%), while the rate of on time, delayed by 1–2 weeks, and delayed by 3–4 weeks for clinical testing was 29.87%, 24.68%, and 15.58%, respectively. Nearly half of the surgery (50.00%) and hospitalization (45.16%) appointments were delayed by 1–2 weeks.

## 4. Discussion

### 4.1. Findings

This study investigated patterns of health services utilization during the first wave of the COVID-19 pandemic in China, using an online patient survey. 52.00% of the overall respondents reported affected health services utilization, with clinical testing and drug purchase being the most affected types. Higher education level, being married, having chronic disease, frequently visiting a community health center or provincial medical institution, spending more time browsing pandemic-related information, perception of high-risk of infection, perception of large health impact of the pandemic, and anxiety/depression during the pandemic were among significant predictors for reporting affected health services utilization. Specially, respondents with chronic disease reported higher degree of health services utilization interruption (62.18%), especially for drug purchase. Outpatient and inpatient services among chronic diseases respondents were postponed to different degrees, along with a decrease in outpatient-related expenses.

### 4.2. Interpretation

Our findings are consistent with existing studies on the health services utilization during the first wave of the pandemic in China and other countries, despite possible differences in the magnitude of services disruption. Hung et al. [7] found that 30.4% of the respondents reported avoidance of medical consultations during the first peak rise of the pandemic in Hong Kong, China. Their reported level of decline was lower than ours, probably because they only focused on complete avoidance of medical visits. They also found that being married, completing tertiary education, and reporting a large impact of the pandemic on mental health were significantly associated with avoiding medical consultation [7]. Zhang et al. [21] found that the total healthcare expenditure and health services utilization declined by 37.8% and 40.8% post-Spring Festival of 2020 compared to the same period of 2019. Moreover, Xiao et al. [20] reported 51% reduction in health facility visits and 49% reduction in inpatient visits across all levels of health facilities in mainland China during February 2020. They found the reductions in both health facility visits and inpatient volume were greater in hospitals than in primary health care facilities and greater in developed regions than in underdeveloped regions, which was consistent with our results that respondents frequently visiting provincial medical institutions reported a higher impact rate. Nationwide routine health information system data was used in this study, and our results could act as a supplement of evidence from the demand-side. For studies from other countries, Wright et al. [10] found that rates of clinical testing fell by 81–90% and new drug therapy by 52–60% for cardiovascular diseases and diabetes, in two large healthcare institutions of the USA during February to March 2020. Similarly, our results also demonstrated largest influence on clinical testing and drug therapy. A systematic review reported 42% reduction in medical visits globally as of August 2020, with greater reductions among people with less severe illness [24]. We also found greater disruptions in health services for the most common health problems including cold or fever, oral diseases, and digestive system diseases.

A number of reasons could explain the decline in health services utilization during the pandemic, which can give insights for possible countermeasures. The fear of infection and lack of knowledge of the virus would influence health seeking behaviors of the public [25,26]. COVID-19 related concerns were also found to be significantly associated with affected health services utilization in our logistic regression analysis. Public information and health messages that reinforce the importance of timely seeking care for serious conditions are needed [25]. The temporary closure or capacity-reduction of health facilities were also important reasons for service disruption. Some elective or preventive health services may be delayed to reduce the risk of transmitting the virus, especially for non-communicable diseases and non-emergency surgeries [5,25,26,27]. Movement restrictions during the peak rise of the pandemic would also affect health services utilization [5,28]. Furthermore, the decrease in income and employer-based health insurance coverage due to work suspension or unemployment may also raise the barriers to accessing health services and exacerbated the pre-existing inequalities in this aspect [29,30,31,32].

To mitigate the impact on health services utilization, several strategies have been implemented during the first wave of the pandemic in China. First, an “extended prescriptions” policy was implemented in February 2020, which allowed health facilities to increase the overall dosage of common drugs in a single prescription based on the evaluation of the health providers, to reduce the risk of infection from less medical visits [33,34]. Specially, for patients with chronic diseases, such as hypertension or diabetes, the prescription could be extended to the overall dosage for three months [33,34]. Second, the novel approach of telehealth was highly encouraged during the pandemic [35], and the medical expenses of the internet-based follow-up health services for common or chronic diseases could be reimbursed by basic medical insurance [36]. Third, the National Health Commission launched the pilot guideline for primary healthcare facilities to provide essential health services for the elderly and chronic diseases patients during the pandemic, including using internet-based platforms, extended prescription, and home-based care [37]. Last, several targeted actions, such as the “Chronic Disease Care Plan”, were launched by a number of internet-based and pharmaceutical companies to meet the essential drug needs for patients with chronic diseases during the peak rise of the pandemic [38,39]. These countermeasures alleviated the impact on essential health services utilization during the first wave of the pandemic in China.

Besides the pattern in the early stage of the pandemic, the evolution of health services utilization should also be investigated due to the rapidly changing nature of the virus. After entering the normalization stage of the COVID-19 prevention and control, the restoration of essential health services was promoted at all levels of health facilities across China since March 2020 [40]. Accordingly, the health services utilization level has gradually increased since then, while it has yet to return to pre-pandemic levels as of April 2020 [21]. Another study also confirmed that outpatient and inpatient visits rebounded after March 2020, but did not recover to the pre-COVID 19 level till June 2020 in China [20]. Furthermore, the second round of the “pulse survey“ by WHO revealed that countries reported generally decreased magnitude of service disruption, while around 90% of countries still reported one or more disruptions to essential health services as of February 2021 [41]. As COVID-19 continues, the strategies mentioned above are further adapted to ensure essential health services utilization in China, while more updated analysis of health services utilization pattern for the Chinese setting is lacking, especially after the emergence of the Omicron variant.

### 4.3. Strengths and Weaknesses

To the best of our knowledge, this was the first large-scale patient survey to investigate health services utilization in both general population and population with chronic diseases in China during the first wave of the COVID-19 pandemic. Through a self-administrated questionnaire, individual-level data were obtained to complement the current evidence base, which mainly consists of aggregated data from the health facility level. The most affected types of health services and significant predictors for service disruption identified in this study, can inform strategies to mitigate the impact of possible epidemics on health services utilization in the future.

We recognize a series of limitations. First, the method of web-based survey and voluntary sampling may introduce some selection bias. People with lower educational attainment and family income may have limited access to the online platform, and people with more health services utilization issues were more likely to attend. Moreover, the voluntary response sample is largely based on ease of access and the authors’ networks. Although we advertised the survey widely using social networking platforms with high penetrance rate in China (e.g., WeChat), more respondents were from the north part of China and the representativeness of the sample was relatively limited. Second, recall bias may exist for participants when answering questions about health services utilization before and during the pandemic. We have assessed the validation of each questionnaire and ruled out those with arbitrary answers and missing responses. Finally, we were unable to capture the long-term trend or resumption of the health services utilization after the first wave of the pandemic in the current study. As the COVID-19 pandemic continues, the adjustment of primary countermeasures from non-pharmaceutical to pharmaceutical interventions, the improvement of accuracy and efficiency of testing, the advent of effective vaccines and treatments, together with the enhanced awareness and knowledge of the virus among the general public, may lead to a reduced impact on essential health services utilization [42]. Therefore, dynamic evaluation is needed to understand how health services utilization among the affected population evolves as the pandemic continues, especially after the emergence of the Omicron variant. Investigation of health services utilization under more recent stages of the COVID-19 pandemic is also needed.

## 5. Conclusions

Affected health services utilization was reported during the first wave of the COVID-19 pandemic in China, especially for those with higher education level, chronic disease, and COVID-19 related concerns. The most significant impact was seen in clinical testing and drug purchase. Enhancing primary healthcare, use of innovative technology of telehealth, extended prescription, together with public communication were countermeasures undertaken by China during the peak rise period to maintain essential health services utilization. As the pandemic progresses, the changing disease characteristics, the adapted health system, along with the enhanced public awareness/knowledge, may lead to changes in the health services utilization pattern. Therefore, further investigation of the health services utilization is needed in China, especially after the emergence of the Omicron variant. In this process, the strategies undertaken during the first wave of the pandemic should be adapted and tailored to different stages of the pandemic.

## Figures and Tables

**Table 1:** Demographic characteristics and health services utilization of the study population.

	All Respondents (N = 4744)		Respondents Reporting No Impact (N = 2277)		Respondents Reporting Impact (N = 2467)		z	*p*
	Frequency	Percentage	Frequency	Percentage	Frequency	Percentage		
Age (Mean ± SD)	31.88	11.44	32.06	11.63	31.72	11.25	0.504	0.615
Gender							1.103	0.294
Male	2319	48.88%	1095	47.22%	1224	52.78%		
Female	2425	51.12%	1182	48.74%	1243	51.26%		
Education level							9.973	0.019 *
Primary school or below	49	1.03%	32	65.31%	17	34.69%		
Middle high school	499	10.52%	228	45.69%	271	54.31%		
Senior high school	1256	26.48%	578	46.02%	678	53.98%		
Undergraduate or above	2940	61.97%	1439	48.95%	1501	51.05%		
Employment status							5.643	0.060
Employed	3072	64.76%	1446	47.07%	1626	52.93%		
Retired	122	2.57%	52	42.62%	70	57.38%		
Non-employed	1550	32.67%	779	50.26%	771	49.74%		
Marital status							5.900	0.015 *
Non-married	2070	43.63%	1035	50.00%	1035	50.00%		
Married	2674	56.37%	1242	46.45%	1432	53.55%		
Household registration								
Urban	2804	59.11%	1302	46.43%	1502	53.57%	6.718	0.010 *
Rural	1940	40.89%	975	50.26%	965	49.74%		
Annual household income in 2019 (RMB)							2.579	0.631
≤50,000	1098	23.15%	531	48.36%	567	51.64%		
50,000–100,000	1483	31.26%	687	46.33%	796	53.67%		
100,000–200,000	1305	27.51%	640	49.04%	665	50.96%		
200,000–500,000	605	12.75%	294	48.60%	311	51.40%		
>500,000	253	5.33%	125	49.41%	128	50.59%		
Annual household medical expenses in 2019 (RMB)							23.139	0.000 ***
≤1000	1234	26.01%	654	53.00%	580	47.00%		
1000–10,000	2435	51.33%	1158	47.56%	1277	52.44%		
10,000–50,000	841	17.73%	370	44.00%	471	56.00%		
50,000–100,000	163	3.44%	67	41.10%	96	58.90%		
>100,000	71	1.50%	28	39.44%	43	60.56%		
Chronic disease							19.677	0.000 ***
No	4313	90.91%	2114	49.01%	2199	50.99%		
Yes	431	9.09%	163	37.82%	268	62.18%		
Frequently visited medical institution							6.283	0.280
Township health centre	719	15.16%	367	51.04%	352	48.96%		
Community health centre	783	16.51%	363	46.36%	420	53.64%		
County-level medical institution	1232	25.97%	594	48.21%	638	51.79%		
Municipal medical institution	1310	27.61%	636	48.55%	674	51.45%		
Provincial medical institution	686	14.46%	312	45.48%	374	54.52%		
Other medical institution	14	0.30%	5	35.71%	9	64.29%		
Time to browse information related to the pandemic per-day							62.115	0.000 ***
Irregular	771	16.25%	444	57.59%	327	42.41%		
Less than 1 h	819	17.26%	401	48.96%	418	51.04%		
1–2 h	2124	44.77%	1030	48.49%	1094	51.51%		
More than 3 h	1030	21.71%	402	39.03%	628	60.97%		
Perception of COVID-19 infection risk							252.629	0.000 ***
Very low	933	19.67%	572	61.31%	361	38.69%		
Fairly low	1784	37.61%	979	54.88%	805	45.12%		
Slightly high	859	18.11%	357	41.56%	502	58.44%		
Fairly high	672	14.17%	242	36.01%	430	63.99%		
Very high	496	10.46%	127	25.60%	369	74.40%		
Perception of the health impact of the pandemic							690.549	0.000 ***
Nearly no	1944	40.98%	1354	69.65%	590	30.35%		
Some	2043	43.06%	773	37.84%	1270	62.16%		
Significant	757	15.96%	150	19.82%	607	80.18%		
Anxiety/depression during the pandemic							266.151	0.000 ***
No	2311	48.71%	1366	59.11%	945	40.89%		
Occasional	1991	41.97%	808	40.58%	1183	59.42%		
Frequent	442	9.32%	103	23.30%	339	76.70%		

SD, standard deviation. * *p* < 0.05, ** *p* < 0.01, *** *p* < 0.001.

**Table 2:** Results of Logistic regression analysis on factors associated with reporting affected health services utilization.

Variables	OR	SE	z	*p* > z	95% CI	
Age	0.99	0.004	−1.250	0.210	0.99	1.00
Gender (Reference: Male)						
Female	0.99	0.066	−0.220	0.823	0.86	1.12
Education level (Reference: Primary school or below)						
Middle high school	2.42	0.828	2.570	0.010 *	1.23	4.73
Senior high school	2.17	0.737	2.280	0.023 *	1.11	4.22
Undergraduate or above	2.06	0.702	2.120	0.034 *	1.06	4.02
Employment (Reference: Employed)						
Retired	1.15	0.264	0.590	0.557	0.73	1.80
Non-employed	0.97	0.080	−0.370	0.708	0.82	1.14
Marital status (Reference: Non-married)						
Married	1.28	0.114	2.820	0.005 **	1.08	1.53
Household registration (Reference: Urban)						
Rural	0.89	0.068	−1.570	0.117	0.76	1.03
Annual household income in 2019 (Reference: ≤50,000 RMB)						
50,000–100,000	0.99	0.093	−0.150	0.881	0.82	1.19
100,000–200,000	0.84	0.087	−1.720	0.085	0.68	1.02
200,000–500,000	0.91	0.116	−0.700	0.483	0.71	1.17
>500,000	0.93	0.161	−0.420	0.674	0.66	1.31
Annual household medical expenses in 2019 (Reference: ≤1000 RMB)						
1000–10,000	1.10	0.089	1.160	0.245	0.94	1.29
10,000–50,000	1.06	0.112	0.540	0.586	0.86	1.30
50,000–100,000	1.17	0.226	0.800	0.426	0.80	1.70
>100,000	1.07	0.304	0.230	0.822	0.61	1.86
Chronic disease (Reference: No)						
Yes	1.27	0.155	1.980	0.048 *	1.00	1.62
Frequently visited medical institution (Reference: Township health centre)						
Community health centre	1.29	0.151	2.180	0.029 *	1.03	1.62
County-level medical institution	1.18	0.128	1.520	0.129	0.95	1.46
Municipal medical institution	1.10	0.125	0.870	0.387	0.88	1.38
Provincial medical institution	1.34	0.180	2.180	0.029 *	1.03	1.74
Other medical institution	2.63	1.635	1.560	0.119	0.78	8.90
Time to browse information related to the pandemic per-day (Reference: Irregular)						
Less than 1 h	1.33	0.148	2.530	0.011 *	1.07	1.65
1–2 h	1.09	0.104	0.940	0.346	0.91	1.32
More than 3 h	1.34	0.146	2.730	0.006 *	1.09	1.66
Perception of COVID-19 infection risk (Reference: Very low)						
Fairly low	1.15	0.104	1.600	0.110	0.97	1.38
Slightly high	1.43	0.153	3.320	0.001 **	1.16	1.76
Fairly high	1.63	0.190	4.220	0.000 ***	1.30	2.05
Very high	2.09	0.287	5.370	0.000 ***	1.60	2.73
Perception of the health impact of the pandemic (Reference: Nearly no)						
Some	3.14	0.228	15.720	0.000 ***	2.72	3.62
Significant	6.24	0.716	15.960	0.000 ***	4.98	7.82
Anxiety/depression during the pandemic (Reference: No)						
Occasional	1.39	0.098	4.670	0.000 ***	1.21	1.59
Frequent	1.80	0.243	4.330	0.000 ***	1.38	2.34

CI, confidence interval; OR, odds ratio; SE, standard error. * *p* < 0.05, ** *p* < 0.01, *** *p* < 0.001.

**Table 3:** Outpatient medical expenses for respondents with chronic diseases (RMB).

	N	November to December 2019	January to February 2020	Z	*p*
Total medical expenses	236	1975 (500–5000)	1000 (300–3000)	5.061	0.000 ***
Out-of-pocket medical expenses	236	500 (200–2000)	412 (100–1300)	4.372	0.000 ***
Drug expenses	236	600 (300–2000)	500 (100–1000)	4.508	0.000 ***
Out-of-pocket drug expenses	236	300 (100–1000)	228 (50–790)	2.760	0.006 **

Expenses were presented as median (interquartile range). * *p* < 0.05, ** *p* < 0.01, *** *p* < 0.001.

## Data Availability

Relevant anonymized data used for this analysis will be made available upon reasonable request to the authors. Requests for data can be made to the corresponding author.
